# Current Status and Applications for Hydraulic Pump Fault Diagnosis: A Review

**DOI:** 10.3390/s22249714

**Published:** 2022-12-11

**Authors:** Yanfang Yang, Lei Ding, Jinhua Xiao, Guinan Fang, Jia Li

**Affiliations:** 1 School of Transportation and Logistics Engineering, Wuhan University of Technology, Wuhan 430063, China; 2 Naval Submarine Academy, Qingdao 266071, China

**Keywords:** hydraulic pump, fault diagnosis, fault prediction, remaining service life prediction, health status monitoring

## Abstract

To implement Prognostics Health Management (PHM) for hydraulic pumps, it is very important to study the faults of hydraulic pumps to ensure the stability and reliability of the whole life cycle. The research on fault diagnosis has been very active, but there is a lack of systematic analysis and summary of the developed methods. To make up for this gap, this paper systematically summarizes the relevant methods from the two aspects of fault diagnosis and health management. In addition, in order to further facilitate researchers and practitioners, statistical and comparative analysis of the reviewed methods is carried out, and a future development direction is prospected.

## 1. Introduction

Hydraulic systems are applied to all crucial mechanical equipment and play an irreplaceable role in the field of industrial production and manufacturing [1]. As the “heart” of the hydraulic system, the hydraulic pump is responsible for converting mechanical energy into hydraulic energy and providing pressure oil for the system [2]. With the development of the hydraulic industry, the structure of hydraulic pumps becomes more and more complex, and the probability of failure also increases; When it breaks down, it may cause the equipment controlled by the system to shut down for a long time, thus reducing the efficiency of the production process, bringing economic and safety problems, and even causing casualties in serious cases [3]. Therefore, it is of great practical significance to make reasonable and accurate fault diagnoses for hydraulic pumps; Under the premise of fault diagnosis, fault prediction, remaining service life prediction and health state detection can further master the safety of the hydraulic pump in operation, which is more conducive to improving the flexibility of the system, so as to prevent the occurrence and development of catastrophic faults in industrial systems, resulting in major losses.

The fault diagnosis method of hydraulic pumps mainly uses different sensors to collect different kinds of state monitoring signals of the hydraulic pump to analyze and reflect the change in the operating state of a hydraulic pump [4]. These state monitoring signals mainly include vibration signals [5], temperature signals [6], flow signals [7], and pressure signals [8], but other signals that can characterize the change of the operating state of the hydraulic pump also belong to the state monitoring signals [9]. Hydraulic pump fault diagnosis methods mainly include signal processing methods [10] and artificial intelligence methods [11], as well as mechanism analysis-based diagnosis methods [12]. The structural composition and operation mechanism of the hydraulic pump is complex, so it is difficult to quantitatively diagnose the fault under the mechanism analysis method. In different operating states of the hydraulic pump, the state monitoring signals present different information, and it is feasible to diagnose faults according to the information presented by the monitoring signals. With the development of artificial intelligence, fault diagnosis can be carried out by analyzing the signal data information when the operating mechanism of the hydraulic pump is fuzzy. In the process of fault diagnosis, there are two crucial problems: one is which state monitoring signals are selected as characteristic signals. The second is how to build a fault diagnosis model. On the premise of fault diagnosis, the “fault threshold” of various faults is extracted, and early fault prediction, remaining service life prediction, and health state detection can be carried out for the hydraulic pump. In view of the above problems, more and more research and investigations have been conducted in recent years, but there is a lack of a timely summary of the developed methods. The purpose of this paper is to provide the latest research progress and application.

**Figure 1:**
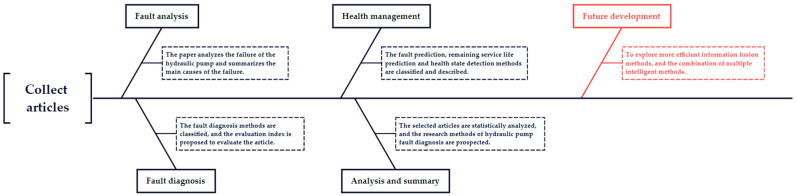
The research process of the paper.

This paper takes the hydraulic pump as the research object and analyzes the application and development of hydraulic pump fault diagnoses in recent years. Collate the articles on fault diagnosis and health management of various hydraulic pumps, and analyze and summarize the articles; Summarize the main causes of hydraulic pump failure; The methods used for fault diagnosis of hydraulic pumps are classified, and the paper evaluation index is proposed to evaluate the selected articles; The methods used for fault prediction, remaining service life prediction and health state detection of hydraulic pumps are described; Finally, the selected articles are statistically analyzed, and the research prospect of hydraulic pump fault diagnosis is given. The research flow of this paper is shown in Figure 1.

This paper is structured as follows. Section 1 explains the importance and challenges of hydraulic pump fault diagnosis for application. Section 2 introduces the research on hydraulic pump faults in published papers and summarizes the fault types. Section 3 proposes the classification scheme of hydraulic pump diagnosis methods and summarizes the application of these methods. Section 4 briefly mentions the research and application of health management of hydraulic pumps. Section 5 makes a statistical analysis of the published papers and outlines future research trends. Section 6 gives a summary of this paper.

## 2. Fault Analysis of Hydraulic Pump

According to the different structures, hydraulic pumps can be divided into gear-type hydraulic pumps, vane-type hydraulic pumps, plunger-type hydraulic pumps, and screw-type hydraulic pumps. Although the components of various hydraulic pumps are different, their oil supply principle is the same, and they all belong to positive displacement hydraulic pumps. Its working principle is essentially the change of the sealing volume, that is, the oil is sucked by the local vacuum formed by the gradual increase of the sealing volume on the side of the oil inlet of the hydraulic pump, and the oil is squeezed into the hydraulic system by the gradual decrease of the sealing volume on the side of the oil outlet.

After a certain period of normal operation of the hydraulic pump, its parts and components will be gradually worn and damaged, or when the hydraulic pump operates under abnormal conditions, various fault phenomena such as increased noise, increased vibration, and decreased flow will occur. The failure of the hydraulic pump may be caused by excessive wear or damage to certain parts in the structure of the hydraulic pump, so the failure of the whole hydraulic pump can be studied from the study of certain parts.

In the hydraulic pump, the rotation of the shaft drives the operation of the whole medium, so the shaft of the hydraulic pump is one of the research contents. Xu et al. [13] analyzed the cause of the driving shaft fracture by calculating the radial force of the driving shaft of the hydraulic gear pump and used the finite element analysis software Ansys to simulate and verify the correctness of the fault cause. Xiao et al. [14] used the life acceleration experiment to analyze the deterioration and failure of the shaft of the hydraulic gear pump, checked the static strength of the broken part, and analyzed the main reasons for the failure of the shaft. Shawkis et al. [15] analyzed the annular crack on the drive shaft of the high-pressure hydraulic screw pump and concluded that one of the reasons for the shaft fracture was fatigue caused by misalignment during the rotation bending process. Xu et al. [16] believed that the main reason for the fracture was the increase of rotation and bending load caused by low viscosity medium through the analysis of macro morphology and microstructure, chemical composition, fracture metallography, and pump operation. Through the metallographic and fracture analysis of different parts of the hydraulic pump shaft, Yordanov B. et al. [17] can see the mixed characteristics in the morphology of the damaged surface, and conclude that the oxidation of the shaft surface and the intergranular corrosion at the grain boundary are one of the reasons for the crack generation and fracture propagation.

In the hydraulic pump, there are faults caused by other parts and hydraulic oil. Li et al. [18] analyzed the mechanics and microstructure of the broken pump housing of the hydraulic gear and found the main reason for the failure of the pump housing. Sekercioglu T. et al. [19] used hardness, chemical analysis, and metallographic examination to analyze the broken gear of the hydraulic gear pump, carried out geometric analysis of the gear of the hydraulic gear pump, and obtained the reason for the fracture of the gear of the hydraulic gear pump. Pflum et al. [20] used the pressure sensor to detect the detection signal in the narrow band frequency domain to analyze the spalling of the mechanical bearing of the hydraulic pump and the failure of the hydraulic gear pump. Hemati et al. [21] used signal processing technologies such as mechanical spectrum, envelope spectrum, and acceleration spectrum to conduct vibration analysis and signal processing of the hydraulic gear pump, and studied the failure of the hydraulic gear pump caused by the looseness of the bearing bush. Lee et al. [22] analyzed the characteristics of hydraulic oil, calculated the friction heat value, and analyzed the phenomenon that caused the failure to study the cause of the failure of the pilot check valve of the hydraulic pump caused by hydraulic oil pollution and leakage. Wang et al. [23] conducted the vibration fatigue test of the flameproof housing of the hydraulic pump regulator and analyzed the factors that caused the housing failure.

In addition to single-component failures, there are also some combined failures. By analyzing the structure and working principle of the external gear hydraulic pump, Zhang et al. [24] analyzed the failure of the external gear pump and proposed corresponding failure solutions. Das et al. [25] analyzed the microscopic cause of rapid wear of hydraulic pumps from the influence of the microstructure of hydraulic gear pump on the corrosion wear behavior of materials. Jiang et al. [26] carried out detailed statistics on various failures of screw pumps to analyze the failure modes of hydraulic screw pumps. Milović et al. [27] took the damage of the high-pressure three-screw oil pump in the regulating oil of the hydropower station as an example to analyze the failure of screw pump wear, thread tear, and filter blockage. Shang et al. [28] analyzed the failure and main causes of hydraulic pump damage and proposed corresponding effective solutions. Hidayath et al. [29] comprehensively considered the hydraulic pump failure caused by hardware and hydraulic oil. UłAnowicz et al. [30] established a simplified three-dimensional solid model of the cylinder piston assembly and gave the piston cylinder block, the inclination adjustment mechanism of the axial-flow hydraulic pump, and the fracture load model of the selected components of the pump, and discussed the actual damage of the axial piston pump.

When studying hydraulic pump faults, there are methods based on mechanism analysis and modeling, software simulation, signal fusion, and artificial intelligence. Fabiś-Domagała et al. [31] proposed the method of combining FMEA matrix analysis and Error Diagram to analyze the fault of the hydraulic gear pump and find out the factors causing the fault. Major et al. [32] proposed the fatigue failure finite element model of screw pump for the most serious fatigue fracture failure of a reciprocating screw of screw pump and carried out model simulation in Ansys. Ma et al. [33] established a simulation model of the hydraulic system by using AMESim software and analyzed the failure modes and mechanisms of key components in the system and their failure effects. Lee et al. [34] proposed to use FMECA to carry out extensive fault analysis of hydraulic gear pumps and proposed to use MFCC combined with a random forest classifier (RFC) to extract features and identify faults of vibration signals.

For the hydraulic pump failures studied in the above literature, it is concluded that the main reason for the failure of the hydraulic pump is the wear of the hydraulic pump. The wear of the hydraulic pump is divided into the situations shown in Table 1.

## 3. Failure Diagnosis Method

The idea of hydraulic pump fault diagnosis based on condition monitoring signals is to collect the condition monitoring signals by sensors, then use signal processing methods to pre-process the collected status monitoring parameters, and then combine the fault diagnosis model to diagnose faults. In this investigation, based on the correct signal acquisition process, the hydraulic pump fault diagnosis methods are divided into the following three categories:

(1) Fault diagnosis based on a single signal;
(2) Fault diagnosis based on multi-signal;
(3) Other diagnostic methods.

### 3.1. Fault Diagnosis Based on Single Signal

At present, among the fault diagnosis methods based on a single signal, the vibration signal is the most widely used condition monitoring signal as the feature input of the fault diagnosis model. This is because once the internal parts of the pump fail, it usually causes changes in the characteristics of the load state structure and other characteristics, so the vibration response of the pump structure will change. Through the measurement of structural vibration signals, and relying on the principle of signal analysis, specific fault information is extracted, and the fault diagnosis is realized by artificial intelligence or signal analysis. Additionally, a few are based on other types of state monitoring signals such as sound signals. In the methods of hydraulic pump fault diagnosis, there are two main categories: the method of hydraulic pump fault diagnosis based on signal processing and the method of hydraulic pump fault diagnosis based on artificial intelligence.

#### 3.1.1. Fault Diagnosis Based on Vibration Signal

(1) Method based on signal processing

The vibration signal has been proven to be useful for fault diagnosis of hydraulic pumps, but it contains noise, interference, and other information without fault characteristics. Therefore, it is necessary to use effective signal processing methods to extract available fault information from vibration signals. The following article has conducted some research on noise removal of vibration signals.

Yu et al. [35] proposed an EWT-VCR fusion method based on EWT and VCR to deal with the nonlinear, multi-frequency, and noise data of vibration signals. Jiang et al. [36] used the method of combining EEMD and PCC to denoise the collected hydraulic pump vibration signals, converted the denoised data into snowflake images by using the symmetric polar coordinate method, and converted the obtained images into gray level co-occurrence matrix, and used the fuzzy c-means algorithm for fault diagnosis. In view of the problem that the vibration signal of the hydraulic pump will be polluted by stronger Gaussian and non-Gaussian noise, Zheng et al. [37] proposed using PSE to extract fault information, effectively highlighting fault features and suppressing noise pollution. Wang et al. [38] studied the DCT denoising method and the CNC denoising method in view of the serious noise problem in the vibration signal of the hydraulic pump. Finally, CNC denoising was adopted, and then HHT was used to extract the fault information of the signal. In order to reduce noise and other interference, Sun et al. [39] carried out local feature scale decomposition for high-frequency harmonic correction of vibration signals and proposed discrete cosine transform high-order spectrum analysis algorithm to extract singular entropy as the degradation feature of hydraulic pumps. Liu et al. [40]. proposed a new rough set fault diagnosis algorithm for hydraulic pumps guided by PCA, aiming at the characteristics of fuzzy fault features and low signal-to-noise ratio of hydraulic pumps, using WA for noise reduction processing, extracting effective fault features, using PCA method for dimensionality reduction and decoupling correlation analysis of these features, using rough set theory to establish a knowledge base of diagnosis rules. Hou et al. [41] proposed a WPD-based denoising method for hydraulic pump fault feature extraction to solve the problem that the feature signal is weak and covered by noise. Wang et al. [42] introduced the idea of WNC denoising in view of the problems of the DCT denoising method, proposed a CNC denoising method, and extracted fault features from the output signal by HHT, effectively solving the problem of missing vibration signal components.

Under the actual conditions, the fault information of hydraulic pumps is still relatively poor, so it is necessary to solve the problem of fault diagnosis under the condition of poor information. Jia et al. [43] proposed a fault diagnosis method based on SPIP and HMM in order to realize fault diagnosis in the case of poor information. This method converts vibration signals into symbol sequences as feature sequences of hidden Markov models, uses genetic algorithms to optimize the symbol space division scheme, and then uses hidden Markov models for fault diagnosis. In view of the shortage of single-scale arrangement entropy when measuring the complexity of vibration signals on a single scale, Wang et al. [44] proposed an MPE entropy value and MMPE. The analysis results of the measured vibration signals of hydraulic pumps verified the effectiveness and superiority of this index as a fault feature of hydraulic pumps. Aiming at the problem of poor detection of fault signals of the hydraulic pump in the early stage, Yu et al. [45] proposed a method of using EWT to decompose the vibration signals of three channels, then defining VCR to divide the weights of components to form a single signal, and using HT to demodulate the characteristic frequency to achieve fault detection of the hydraulic pump. Deng et al. [46] proposed a fault diagnosis method based on EMMD and Teager energy operator demodulation to solve the problem of weak early fault vibration signals of the hydraulic piston pump.

In the process of feature extraction of vibration signal, the original primary method has some limitations, so it needs to be improved. Zheng et al. [47] proposed an IEWT-based signal processing method for hydraulic pump fault diagnosis in view of the serious over-decomposition problem of EWT. Jiang et al. [48] proposed a method of hydraulic pump fault signal demodulation based on LMD and IAMMA. Li et al. [49] proposed a hydraulic pump fault feature extraction method based on MCS and RE. According to the maximum relational entropy criterion and the progressive fusion strategy, a relative entropy algorithm was established to fuse the initial features into new degraded features.

Some comparison methods and processing of vibration signals from different angles can still play a role in fault diagnosis of hydraulic pumps. Gao et al. [50] compared and analyzed the two fault diagnosis methods of WT and spectrum analysis, and concluded that when analyzing the same vibration signal dataset, the diagnosis ability of the method based on WT was more accurate. Sun et al. [51] proposed a fault diagnosis method for hydraulic pumps based on a fusion algorithm that processes vibration signals successively through LCD and DCS to improve the characteristic performance of signals. Siyuan et al. [52] proposed a hydraulic pump fault diagnosis method based on PCA of Q statistics, which uses normal vibration signals to establish a principal component model and then compares it with the test samples obtained by Q statistics to diagnose faults. Wang et al. [53] proposed a fault diagnosis method based on WP and MTS. This method performs WPT on the collected vibration signals, removes redundant features by the Taguchi method, extracts principal components, and then uses an MD-based calculation method to diagnose hydraulic pump faults. Chen et al. [54] proposed a hydraulic pump fault diagnosis method based on compression sensing theory, which uses the original vibration signal of the hydraulic pump to construct a compression dictionary matrix, uses the Gaussian random matrix to compress the vibration monitoring data of the hydraulic pump and uses a SOMP algorithm to reconstruct the test data. Tang et al. [55] proposed a fault diagnosis method for hydraulic pump fault under variable load in order to solve the problem of dynamic characteristic analysis of hydraulic pumps, which collects vibration signals and uses the axial RMS trend gradient for fault diagnosis.

The fault diagnosis methods of hydraulic pumps based on signal processing have their own limitations, such as time domain analysis, which is easy to cause misjudgment when the fault is serious, has large randomness, and is not suitable for non-stationary signals; Frequency domain analysis cannot reflect time characteristics and is not sensitive to early faults; The multi-sensor information fusion method has some limitations, such as the difficulty of sensor configuration and management, and the complexity of fault information fusion algorithm design.

(2) Methods based on artificial intelligence

Although the signal processing method of vibration signal can effectively extract and express the fault information of hydraulic pumps, the speed and accuracy of its method to diagnose the fault of hydraulic pumps are not ideal. However, with the rapid development of artificial intelligence, more and more intelligent algorithms and models can quickly diagnose faults, and the self-learning ability of artificial intelligence makes the accuracy of diagnosis algorithms and models a high level. Therefore, the artificial intelligence method combining the signal processing method based on vibration signal feature extraction with the artificial intelligence diagnosis algorithm and model is more effective.

➀ Artificial intelligence method based on neural network

With the generalization ability of a neural network, more and more neural network models are applied to fault diagnosis of hydraulic pumps. The fully connected neural network has the ability of self-learning and searching for optimal solutions at high speed. It has the advantages of high accuracy and rapidity in the fault diagnosis of hydraulic pumps. Gao et al. [56] proposed a fault diagnosis method based on EMD and NN. Sun et al. [57] proposed a hydraulic pump fault diagnosis method based on ITD and softmax regression, which uses ITD to process the vibration signal of the hydraulic pump and trains the softmax regression model to diagnose possible fault modes. Ding et al. [58] used LMD to process the collected vibration signal data of the hydraulic pump to form a feature vector, trained the Softmax regression model with the reduced features, and obtained the fault diagnosis model of the hydraulic pump. Jikun et al. [59] proposed a fault diagnosis method for hydraulic pumps based on WPT and SOM-NN. This method uses WPT to extract features from vibration signals, and SOM-NN trains through normal samples and fault samples to diagnose faults when they occur.

Although a fully connected neural network has high accuracy, it needs a lot of trainable variables, which is prone to model overfitting, and model convergence speed needs to be improved. The convolutional neural network can further extract the features of the input through the convolution kernel, and the trainable parameters of the model are greatly reduced by sharing the convolution kernel. Tang et al. [60] proposed an intelligent fault diagnosis method for hydraulic pumps based on CNN and CWT, which uses CWT to convert the original vibration signal into image features, and establishes a new deep convolutional neural network framework that combines feature extraction and classification, and can further improve the convergence speed of the model by optimizing the CNN’s hyperparameters. Zhu et al. [61] proposed an improved AlexNet intelligent fault diagnosis method based on WPA combined with changing the network structure, reducing the number of parameters and computational complexity. Tang et al. [62] proposed a normalized convolutional neural network (NCNN) framework based on a batch normalization strategy for feature extraction, and then used a Bayesian algorithm to automatically adjust the model hyperparameters. BP neural network was used for fault diagnosis based on synchronous noise wavelet transform of vibration signals. Yan et al. [63] proposed a simple 7-layer CNN network setting method based on a base-period to realize fault diagnosis of hydraulic pumps. Zhu et al. [64] improved the core size and number based on the standard LENet-5 model, added a batch normalization layer to the network architecture, and built a PSO-Improve-CNN fault diagnosis model based on vibration signals by automatically optimizing the model’s hyperparameters through PSO. Tang et al. [65] established an adaptive CNN hydraulic pump fault diagnosis model using Bayesian Optimization hyperparameters based on the Gaussian process by taking the time-frequency image of the vibration signal after CWT as input data. Tang et al. [66] converted the vibration signal into an image through CWT, preliminarily extracted effective features from the converted time-frequency image, built a CNN model to achieve fault diagnosis, and realized the visualization of simplified features by using T-DSNE.

In addition, there is also a new neural network model based on the improved functions in the neural network. Luc et al. [67] proposed a CPRBF-NN composed of multiple parallel-connected RBF subnets in combination with chaos theory and applied the proposed method in combination with vibration signals to fault diagnosis of hydraulic pumps. Huijie et al. [68] proposed to integrate the RELU activation function and Dropout strategy into SAE to directly train and identify vibration signals, forming a SAE-based fault diagnosis method for hydraulic pumps. Du et al. [69] proposed a method to extract 17 time-domain features of vibration signals, analyzed the sensitivity of features to the failure to select sensitive feature parameters, built a neural network diagnosis model, and formed a hydraulic pump fault diagnosis method based on sensitivity analysis and PNN. Dongmei et al. [70] took the vibration data as the input and the failure mode matrix as the target output to obtain a PARD-BP-based fault diagnosis method.

➁ Artificial intelligence method based on a support vector machine

Support vector machine (SVM), which originates from statistical learning theory, can be used for supervised learning, unsupervised learning, and semi-supervised learning, and it has an outstanding ability for both linear and nonlinear signals. Casoli et al. [71] collected vibration signals and used them to extract features for fault diagnosis, reduced the obtained features to reduce the amount of calculation, and used them to train different types of support vector mechanisms to build hydraulic pump fault diagnosis models. Tian et al. [72] proposed a fault diagnosis method based on WPT, SVD, and SVM. Lu et al. [73] proposed a new method for hydraulic pump fault diagnosis that combines EEMD and SVR models. This method uses a combination of GA and grid search to optimize the parameters of SVM. Fei et al. [74] proposed a fault extraction method combining WPA, FE, and LLTSA, and then proposed a hydraulic pump fault diagnosis method combining SVM. Niu et al. [75] proposed a hybrid fault diagnosis method for hydraulic pumps that combines the RNS algorithm and SVM. Zhao et al. [76] proposed that CEEMD is used to decompose the signal, then STFT and TFE are used to extract the fault features, and multi-class SVM is used to diagnose the fault of the hydraulic pump. Hu et al. [77] proposed the SS-SVM fault diagnosis algorithm, which constitutes a multi-fault classifier for hydraulic pump fault diagnosis. This method requires only a few fault data samples for training the classifier and has strong fault diagnosis ability in the case of small samples. Tian et al. [78] proposed a degradation feature extraction method for hydraulic pumps based on ILCD and MF, and input the degradation feature into BT-SVM for fault diagnosis of hydraulic pumps.

➂ Artificial intelligence method based on a limit learning machine

In essence, the limit learning machine maps the input feature data to the random space and then uses the least square linear regression. Its advantages are that the hidden layer does not need iteration, the learning speed is fast, and the generalization performance is good. Li et al. [79] proposed a comprehensive fault diagnosis method for hydraulic pumps based on MEEMD, AR spectral energy, and WKELM method. Ding et al. [80] proposed a fault diagnosis method combining EWT, PCA signal processing method, and ELM. Liu et al. [81] proposed a time series dynamic feature extraction method based on CEEMDAN and CMBSE, based on a hydraulic pump fault diagnosis method combining t-SNE and WOA-KELM was proposed. Lan et al. [82] proposed an intelligent fault diagnosis method for hydraulic pumps based on WPT, LTSA, EMD, LMD multiple signal processing technology, and ELM identification technology.

➃ Artificial intelligence method based on fuzzy theory

The structure of the hydraulic pump is complex, and the causes of the failure of the hydraulic pump cannot be completely divided, which has certain fuzziness. Therefore, the fuzzy set and membership function of the hydraulic pump can be constructed, and the fault of the hydraulic pump can be diagnosed using the method of fuzzy theory. Wang et al. [83] proposed a method to capture the degraded characteristic signal of SIE and then used the vibration signal combined with the FCM algorithm to build a hydraulic pump fault diagnosis method. Wang et al. [84] proposed a rough set method for mechanical fault diagnosis, which extracts the spectral features of vibration signals as the attributes of learning samples, and uses a set of decision rules obtained from the upper and lower approximation of decision classes as a rough classifier. Wang et al. [85] extracted diagnostic features from the spectrum of vibration signals, processed the spectrum representing a variety of different fault states using fuzzy membership function, and made fuzzy comprehensive discrimination according to anti-fuzzy diagnostic rules, thus realizing correct diagnosis of different fault spectra. Mollazade et al. [86] studied a new method of hydraulic pump fault diagnosis based on vibration signal PSD combined with DT and FIS.

The method based on a neural network is to extract fault features by signal processing, then use a neural network as the fault diagnosis model, that is, the fault mode analysis after fault signal processing, so as to realize the nonlinear mapping from fault symptoms to fault causes. The diagnosis reasoning process of this method is not clear and the diagnosis explanation is not intuitive. The fuzzy reasoning method is suitable for dealing with uncertain and incomplete information in pump fault diagnosis. Its disadvantage is that it is difficult to establish complete rules and membership functions, and its learning ability is poor.

#### 3.1.2. Fault Diagnosis Based on Other Signals

In addition to the frequent vibration signals, some other condition monitoring signals also contain fault information about the hydraulic pump, and the new monitoring signals are accompanied by new analysis methods, which makes the fault diagnosis methods of the hydraulic pump more diversified. Shengqiang et al. [87] proposed a KPCA fault diagnosis method based on the sound signal, described the feature extraction of the acoustic signal, and used the KPCA method to diagnose the hydraulic pump fault in view of the unsuitable use of the hydraulic pump vibration sensor and the limitations of the fault diagnosis method based on vibration signal processing. Jiang et al. [88] proposed a fault diagnosis method for an axial piston hydraulic pump based on the combination of the MFCC feature extraction method and ELM. The MFCC voiceprint feature of the processed sound signal is extracted from the acoustic signal, and the ELM model is established for fault diagnosis. Based on the standard LeNet, Zhu et al. [89] used PSO to automatically select the hyperparameters of the diagnosis model and built a PSO-CNN hydraulic pump fault diagnosis model with acoustic signals as input.

Tang et al. [90] used CWT to obtain the time-frequency characteristics of the pressure signal, set the initial hyperparameters to establish a deep CNN, and then used the Bayesian optimization method to realize automatic learning of the main important hyperparameters to build an adaptive CNN-based hydraulic pump fault diagnosis method. Wang et al. [91] used FEMD to decompose the pressure signal and then extracted useful fault information from the signal through RE. This method also has a good ability to suppress noise. Liu et al. [92] proposed to use the instantaneous angular speed (IAS) signal obtained by the equal angle method to diagnose the hydraulic pump fault under non-stationary conditions.

The four major wear faults of hydraulic pumps summarized in the literature research are classified as Fault I: friction wear faults; Fault II: abrasive wear fault; Fault III: pit wear fault; Fault IV: corrosive wear fault. In addition, it further evaluates the paper from the following points:

Index I: enhance fault characteristics;
Index II: optimization of fault diagnosis algorithm;
Index III: adapt to strong noise environment;
Index IV: high diagnostic accuracy.

The above four types of faults and four types of evaluation indicators are applicable to this chapter. The application of fault diagnosis based on a single signal is shown in Table 2.

### 3.2. Fault Diagnosis Based on Multiple Signals

The fault information contained in the current single signal processing is limited. In order to increase the collection of fault information, the characteristic signals of multiple signals can contain more and higher dimensional fault information, which is conducive to improving the accuracy of fault diagnosis of hydraulic pumps and introducing more innovative ways for fault diagnosis of hydraulic pumps.

(1) Method based on signal processing

The essence of the multi-signal hydraulic pump fault diagnosis method is to process each input signal separately, and then use a certain fusion method to fuse the feature information contained in the multi signals, so that the extracted fault information is enough to diagnose the fault state. Liu et al. [93] proposed a fault diagnosis method for hydraulic gear pumps based on EEMD and the Bayesian network. This scheme is a method based on multi-source information fusion. Compared with the traditional fault diagnosis method using only EEMD, this method can comprehensively utilize all useful information other than sensor signals. Lu et al. [94] proposed a multi-source information fusion fault diagnosis method based on D-S evidence theory, which uses a fuzzy membership function to construct the basic probability assignment of three evidence bodies. Based on the acceleration, power consumption, flow, and pressure signals under different states, Buiges et al. [95] used the collected signals to compare with the normal state signals for fault diagnosis. Przystupa et al. [96] considered displaying the changes of pressure and flow on FFT and STFT spectrum to realize the application of short-time Fourier transform to fault diagnosis of hydraulic pumps under different operating conditions. Ma Z. et al. [97] established a variable rate inverse gaussian process model to describe the deterioration behavior of the pump, and proposed a Bayesian statistical fault diagnosis method for pressure and flow degradation data analysis. Ruixiang et al. [98] used pressure spectrum signal, temperature signal, and motion signal as diagnostic features, and then used information fusion technology to diagnose hydraulic pump faults. Du et al. [99] proposed a hierarchical clustering fault diagnosis scheme that distinguishes obvious faults through single signal processing of vibration and flow and uses data fusion technology to find fuzzy information. Zengshou et al. [100] proposed an information fusion diagnosis method based on improved D-S evidence theory and space-time domain. Du et al. [101] proposed a clustering diagnosis algorithm based on statistical ARPD in the diagnosis method based on vibration, flow, and pressure signals. Fu et al. [102] studied the relationship between the Bayesian network algorithm and the fault components of the hydraulic pump and then used the Bayesian network algorithm to diagnose the fault when the simulation data of vibration, pressure, temperature, and flow are incomplete.

(2) Methods based on artificial intelligence

Similar to intelligent methods in Section 3.1, the multi-signal hydraulic pump fault diagnosis method is divided into neural network-based method, classifier-based method, and migration learning-based method.

➀ Artificial intelligence method based on neural network

In the structure of neural networks, the number of neurons in the input layer often exceeds one, so the multi-signal input is compatible with the multi-input characteristics of the input layer of the neural network structure.

The convolutional neural network has exceeded the discrimination ability of human eyes in the accuracy of image recognition, so the digital signal of the hydraulic pump can be converted into an image signal for the convolutional neural network to diagnose the fault of the hydraulic pump. Tang et al. [103] proposed an intelligent fault diagnosis method based on the adaptive learning rate of a neural network to diagnose different fault types by using CWT to convert the three original signals of vibration signal, pressure signal, and sound signal into two-dimensional time-frequency images, and using adaptive learning rate strategy to establish an improved deep CNN model. Taking the vibration signals and pressure signals of hydraulic pumps as the analysis objects. Jiang et al. [104] proposed a fault diagnosis algorithm for hydraulic pumps based on EWT and one-dimensional CNN and deployed the one-dimensional CNN model to the cloud platform to achieve real-time fault diagnosis based on the cloud platform. When based on one-dimensional input signals, there is also a high-precision neural network structure to improve the accuracy of hydraulic pump fault diagnosis. An RBF neural network adopts a linear optimization strategy and has fast learning speed and can approach any nonlinear function with arbitrary accuracy. Zuo et al. [105] built a hydraulic pump fault diagnosis method based on RBF neural network, which takes the pump shell vibration signal and pumps outlet pressure pulse signal as input characteristics.

There is also PNN with RBF neural network function, which is a neural network based on Bayesian decision rules. Zuo et al. [106] built a hydraulic pump fault diagnosis method based on PNN, which takes the pump casing vibration signal and pump outlet pressure pulse signal as input characteristics. Dong et al. [107] used WPT to extract the main fault information contained in the power signal in the historical data, combined with the parameters such as force, oil pressure, casing pressure, and dynamic liquid level to build the fault feature vector, established the PNN model, obtained the mapping relationship between the fault feature vector and the fault form through training the model, and diagnosed the fault form to be entered according to the fault feature vector to be entered. Jiao et al. [108] collected vibration signals and pressure signals to establish a fault diagnosis model based on EMD and PNN. Li et al. [109] proposed a hydraulic pump fault diagnosis method based on the combination of kernel principal components and PNN. This method uses KPCA to reduce the dimension of multi-source data and then diagnoses the fault mode through the PNN network.

➁ Classifier based approach

The function of a classifier is to classify chaotic targets into different categories according to different input signals. In the fault diagnosis of hydraulic pumps, the input signal mapped faults can be classified by the classifier to diagnose the faults. Lakshmanan et al. [110] proposed a hydraulic pump fault diagnosis method that takes the pressure signal, flow signal, and torque signal of the pump as original real-time data for feature extraction, and inputs them into SVM after CWT. Jiang et al. [111] used the decision tree to build a random forest model, trained six continuous variables of the hydraulic screw pump system as input characteristics, and built a hydraulic pump fault diagnosis method based on the random forest model. Hu et al. [112] built a multi-fault diagnosis system based on data fusion according to the D-S evidence theory and used DMM to build a fault diagnosis feature with a basic probability assignment function, ensuring the objectivity of reliability distribution evaluation.

➂ Methods based on Transfer Learning

In order to generalize the ability of the model, the trained model parameters can be migrated to the new model to help train, which can make the initialization performance of the model higher, the promotion rate faster, and the convergence better. Miao et al. [113] used CEEMD and SVD to decompose pressure signal, vibration signal, and flow signal to construct feature vectors and built a hydraulic pump fault diagnosis method through a TrAdaBoost migration learning algorithm. He et al. [114] proposed a migration learning algorithm based on deep MFAM and designed a multi-signal fusion module that assigns weights to vibration signals and acoustic signals, improving the dynamic adjustment ability of the method.

The application of multi-signal-based fault diagnosis is shown in Table 3.

### 3.3. Other Fault Diagnosis Methods

Whether it is based on signal processing or artificial intelligence, it is based on the data-driven fault diagnosis method of hydraulic pumps. This method realizes fault diagnosis of a hydraulic pump by using the mapping relationship between digital signal and fault and does not describe the mechanism function of fault in detail. Some studies have proposed new knowledge or concepts based on the relationship between non digital signal information and hydraulic pump fault mapping [115,116,117,118,119]. 

On the basis of an accelerated life test, Guo et al. [120] proposed a dynamic grid technology to simulate the internal flow field of hydraulic pumps in detail. On the basis of film thickness analysis, Ma et al. [121] put forward a hydraulic pump diagnosis method based on elastohydrodynamic lubrication model analysis by comprehensively considering structural parameters, working condition parameters, and material performance parameters. In view of the multi-crack fault of the hydraulic gear pump gear, Zhao et al. [122] established the vibration wavelet finite element calculation formula of complete gear and cracked gear, studied the fault diagnosis of blind source separation and particle swarm optimization algorithm, and correctly diagnosed the location of multiple cracks of the gear.

### 3.4. Centrifugal Pump Fault Diagnosis Method

The above content is mainly a detailed analysis of the fault diagnosis method of the hydraulic pump, and as a centrifugal pump that also transports liquid, it is also of comparative significance to analyze it. In centrifugal pumps, it is necessary not only to identify the fault but also to discover the severity of the failure and classify it. 

Muralidharan et al. [123] used the DWT to calculate the wavelet characteristics of the vibration signal, used rough sets to generate rules, and used fuzzy logic to classify. Sakthivel et al. [124] used the C4.5 decision tree algorithm to extract statistical features from vibration signals in good and fault states for fault diagnosis. Muralidharan et al. [125] studied the vibration-based fault diagnosis method of a monoblock centrifugal pump and found the best wavelet suitable for single-block centrifugal pump fault diagnosis by calculating and comparing. Nagendra et al. [126] used two different machine learning techniques, SVM and ANN, for centrifugal pump fault diagnosis. It was found that the machine learning method based on ANN combined with chi-square and XGBoost feature ranking techniques is superior to the SVM. Wang et al. [127] proposed a centrifugal pump fault diagnosis method based on CEEMD-sample entropy (SampEn) combined with RF. Based on the characteristic evaluation of the information ratio combined with principal component analysis, Ahmad et al. [128] proposed a new Ir-PCA method. The comparison results found the method was superior to existing advanced methods in terms of fault classification accuracy. ALTobi et al. [129] used MLP and SVM to classify the six fault states and normal states of the centrifugal pump. Therefore, an MLP hybrid training method based on the combination of Back Propagation (BP) and Genetic Algorithm (GA) was proposed. 

### 3.5. Fault Diagnosis Block Diagram

**Figure 2:**
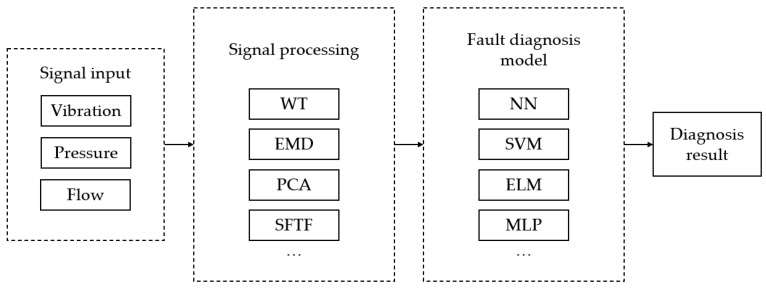
Fault diagnosis block diagram.

Based on the fault diagnosis methods proposed in the above literature, I have summarized the following fault diagnosis block diagram, as shown in Figure 2. Since there are many types of diagnosis methods and many expand on the basic methods, I just list the basic methods for reference.

## 4. Fault Prediction and Health Management

On the basis of fault diagnosis, appropriate prediction and analysis methods can be used to achieve fault prediction. Furthermore, for the health management of the whole life cycle of the hydraulic pump, the remaining service life of the hydraulic pump can be predicted and the whole process of health status monitoring of the hydraulic pump can be studied.

### 4.1. Fault Prediction

To maintain the stable operation of the hydraulic pump in its whole life cycle, the failure prediction of the hydraulic pump can predict the failure that will occur in the early stage of the failure, so as to timely repair the failure in the early stage of low cost and reduce the expansion of loss. The methods of hydraulic pump fault prediction can be roughly divided into two parts, intelligent prediction, and non-intelligent prediction.

The non-intelligent prediction method refers to that the prediction method has no self-learning ability. In short, the non-intelligent prediction method does not use mechanical learning or neural network, which makes the usability of this method relatively weak. Gomes et al. [130] used the empirical model of degradation evolution combined with Kalman filter technology to predict the failure of hydraulic pumps, and successfully predicted two-time series from actual operation to failure data. Amin et al. [131] developed an online health monitoring system for hydraulic pumps by using feature extraction, a fuzzy reasoning system, and knowledge fusion technology. Bykov et al. [132] described the analysis of the state data set of the hydraulic system and tried to diagnose the failure in the valve switching mode, so as to further study the possibility of predicting the failure. Ma et al. [133] analyzed the key failure modes of aircraft hydraulic pumps based on operation and maintenance statistics and proposed a failure prediction method based on multi-source information fusion. Lisowski et al. [134] constructed a function-component matrix (EC) and a component-failure matrix (CF) by using the quality method and then multiplied the two matrices to obtain a function-failure EF matrix containing potential failure information, thus realizing the failure prediction of hydraulic pumps.

Intelligent prediction methods mainly include prediction methods with self-learning ability using neural networks or machine learning. To improve the accuracy of fault prediction, Li et al. [135] proposed a hydraulic pump fault prediction method based on BE and DBN, which is based on the DBN model of constraint limit RBM as a prediction model and introduces QPSO to search the optimal value of the initial parameters of the network. Xu et al. [136] analyzed the cause and mechanism of hydraulic pump degradation due to wear, established a degradation model through joint simulation of Simulink and AMESim, and predicted the failure of the hydraulic pump using a multi-step SVM algorithm. Ding et al. [137] proposed a fault prediction method based on logistic regression that obtains a hydraulic pump fault prediction model by LMD processing of the pump vibration signal, feature reduction using PCA, and training the LR model with the reduced features. Tian [138] used the method of combining EEMD and SEOS to envelope demodulate the vibration signal of the hydraulic pump, and then used WPA to extract the fault features, to establish a hydraulic pump fault prediction model combining WPA and SVM. Sun et al. [139] proposed a multi-channel vibration signal fusion method based on DCS. This method takes the synthetic spectral entropy as the feature and uses the extracted feature to establish an ESN model for prediction, which can be used for fault prediction of hydraulic pumps.

### 4.2. Prediction of Remaining Useful Life

During the normal use of the hydraulic pump, the remaining useful life of the current hydraulic pump can be predicted in time, and the working condition of the hydraulic pump can be adjusted in time through the working time, which is conducive to extending the normal useful life of the hydraulic pump. The remaining useful life prediction methods of hydraulic pumps can be roughly divided into two categories, data-driven methods and model-driven methods.

➀ Data-driven approach

The data-driven methods can be divided into neural network methods and non-neural network methods. Lee et al. [140] constructed HI through vibration signal and pressure signal, and trained a Bi-LSTM neural network using different performance indicators for RUL prediction of hydraulic pumps. Wang et al. [141] used DCAE to characterize the vibration data of hydraulic pumps, constructed HI to determine the degradation state, and input the health index as a tag into the RUL prediction model based on the Bi-LSTM network. Guo et al. [142] used VMD, Hilbert, and FA to process the vibration data of the hydraulic pump, established the degradation evaluation index, trained the Trainbr-RBFNN model with the degradation evaluation index, and obtained the RUL prediction model for the hydraulic pump. 

The non-neural network method can still achieve the RUL prediction of hydraulic pumps. Yu et al. [143] proposed a MAAKR method for information fusion, using 3B-Spline with monotonic constraints to build Hi, and using the MCPF method to monotonically update the random coefficients of the model to achieve RUL prediction of hydraulic pumps. Tongyang et al. [144] proposed an AOPF prediction method to improve the long-term prediction accuracy of RUL and used the MCS method to estimate the posterior probability density function of the future state of the hydraulic pump. Li et al. [145] proposed a new method for RUL prediction of hydraulic pumps based on KPCA and JITL. This method uses WT to extract features, KPCA to fuse features, and constructs an RUL prediction method based on k-VNN and JITL methods.

➁ Model-driven methods

The data-driven method is to use the data information to map the tag of the target fault of the hydraulic pump through the processing and analysis of the data. This method completely bypasses the professional knowledge of the hydraulic pump and only has the mapping relationship from input to output. Based on the model-driven approach, starting from the expertise of hydraulic pumps, mathematical explicit relationships are constructed. Geng et al. [146] proposed a life assessment method that combines SMOTE algorithm, KS test, and cumulative damage theory. The SMOTE algorithm is used to solve the imbalance problem between sample groups, and KS is the classic method for evaluating the goodness of fit. Zhonghaim et al. [147] obtained the fatigue life of the piston by using DLDR through the analysis of the actual load spectrum of the hydraulic piston pump and simulated the fatigue life of the piston by using the finite element analysis software. Wang et al. [148] described the performance degradation model with the Wiener process, predicted the remaining useful life (RUL) of the pump, estimated the initial parameters of the wiener process by MLE using the EM algorithm, estimated the drift coefficient of the wiener process by recursive estimation using Kalman filter method and calculated the RUL of the pump according to the performance degradation model based on wiener process. Wang et al. [149] used the contaminant sensitivity theory of the hydraulic system to derive the mathematical explicit relationship between oil pollution and the useful life of the piston pump and predicted the useful life of the piston pump under certain pollution conditions using a group of experimental data. Sun et al. [150] proposed an improved IG process model to describe the wear degradation of hydraulic pumps and used Monte Carlo integration and EM algorithm to estimate the model parameters. 

### 4.3. Health Status Detection

The real-time health monitoring of the hydraulic pump can diagnose whether the operating state of the hydraulic pump is healthy at each time, which is conducive to the timely adjustment of the hydraulic pump in response to emergencies and the management and use of the hydraulic pump. 

The detection of the health state of the hydraulic pump is not limited to the detection of the fault state, so the amount of data required is very large. A neural network can achieve considerable effect in processing large sample data. According to different health states of hydraulic pumps, Shaowu et al. [151] proposed that after collecting vibration signal data of hydraulic pumps, STFT, WT, and Wigner-Will distributions are used to form time-frequency maps, and then CNN is used to classify and identify time-frequency images of different volumetric efficiencies of hydraulic pumps, so as to monitor the health status of hydraulic pumps. Lin et al. [152] proposed that according to the distribution of the information entropy of the characteristic parameters of the hydraulic pump, various state characteristic parameters can be obtained to characterize the contribution of the hydraulic pump in health, so as to realize the fusion of various characteristic parameters, and then use the grey theory to detect the health state of the hydraulic pump. Hancock et al. [153] researched and developed a method to decompose the vibration signal of vertical hydraulic pumps using WPA, and input the characteristic signal into the adaptive neuro-fuzzy inference fault detection system for pump health state detection. Succi et al. [154] take the fundamental pumping frequency and its harmonics as the input features of the neural network model and use the multilayer neural network model of back propagation and Kohonen feature map to detect the health state of the hydraulic pump. 

There are also some studies that use non-neural network methods, which can also achieve the purpose of detecting the health state of hydraulic pumps. Zhouf et al. [155] proposed a WOA-based RSDD method to extract feature parameters, which combined with the modified hierarchical amplitude aware displacement entropy MHAPE to form a health state detection method for hydraulic pumps. Gao et al. [156] proposed a health diagnosis method for hydraulic pumps based on WPD and WCRA and developed a health detection system based on WPD residual analysis. Shapping et al. [157] used the method of combining WPD and Hilbert envelope demodulation to eliminate the interference effect of radial and axial acceleration signals, replaced Shannon entropy with NE for state identification, and proposed a WPNE-based method for identifying the health state of hydraulic pumps.

## 5. Analysis of the Summary Paper

### 5.1. Statistical Analysis

**Figure 3:**
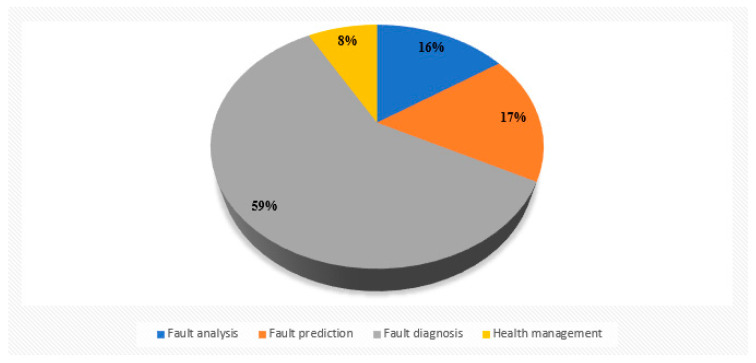
Different research directions of hydraulic pump faults.

Figure 3 shows the statistics of different research directions of hydraulic pump faults in recent years in the literature listed in this paper, and it can be seen that the mainstream research direction is still a fault diagnosis. Equipment fault diagnosis technology has developed to today and has become an independent interdisciplinary comprehensive information processing technology, it is based on reliability theory, cybernetics, information theory, and system theory as the theoretical basis, modern test instruments and computers as a means, combined with the special laws of various diagnostic objects and gradually formed a new discipline, so it is loved by many scholars for research.

**Figure 4:**
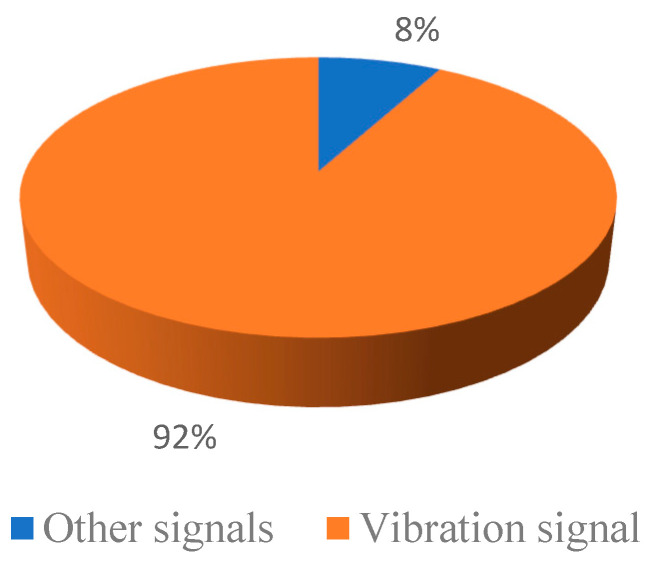
Single signal scale.

Figure 4 shows that among the fault diagnosis methods of hydraulic pumps based on single signals, the fault diagnosis method uses vibration signals to diagnose the faults of hydraulic pumps, which is the first choice for most studies at present. More than 90% of scholars in the selected articles use vibration signals. 

**Figure 5:**
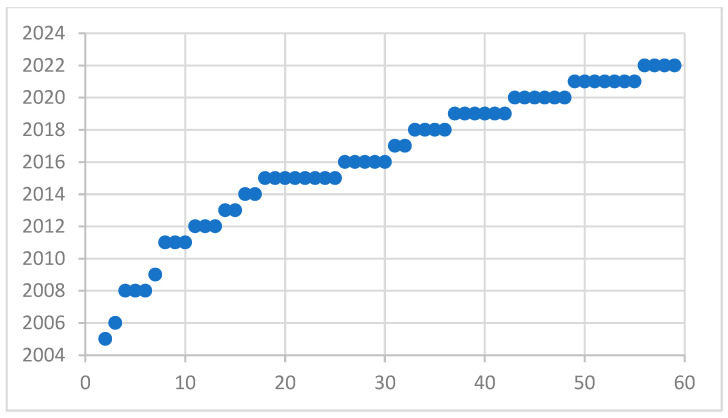
Development trend of single signal articles.

**Figure 6:**
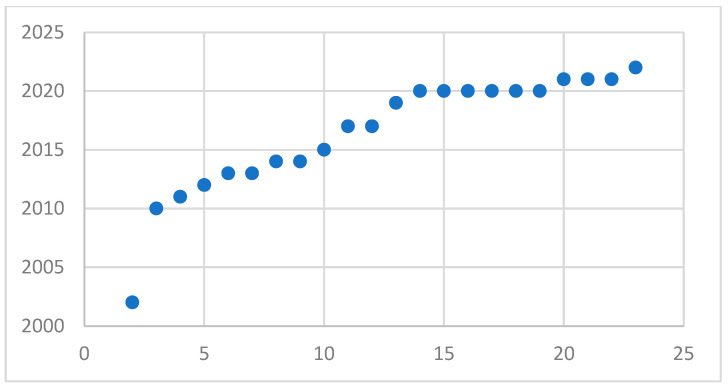
Development trend of multi-signal articles.

With the development of fault diagnosis algorithms in recent years, more and more research on hydraulic pump fault diagnosis has been carried out, which is almost a straight-line trend. As shown in Figure 5 and Figure 6, it can be concluded from the analysis of the two figures that the research on fault diagnosis of hydraulic pumps will continue to increase in the future. With the development of detection signals from simplicity to complexity, it can be seen that the research of single signal fault diagnosis is more than that of multi-signal methods. However, with the development of signal fusion technology, the research of multi-signal fault diagnosis is also increasing year by year.

**Figure 7:**
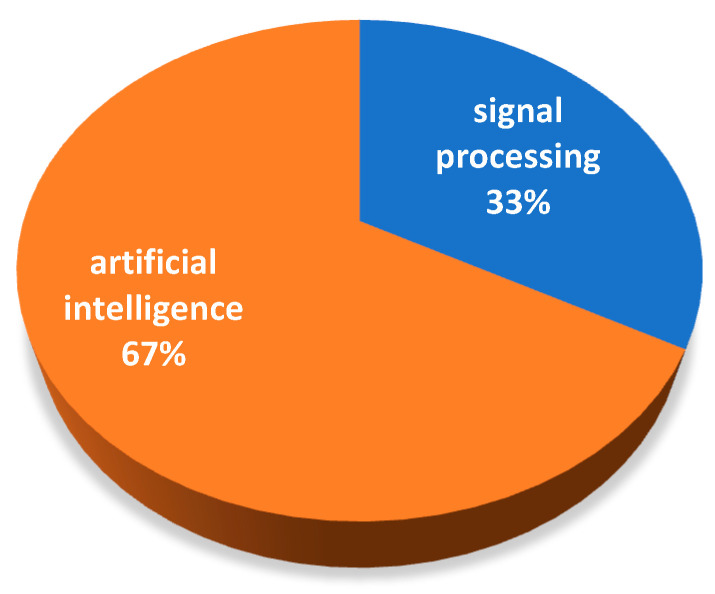
The ratio of signal processing to artificial intelligence.

Figure 7 shows the proportion of signal processing and artificial intelligence, which shows that diagnosis methods based on artificial intelligence are more and more popular. Although the signal processing methods are developing year by year, most of the research focuses on the composite method of signal processing methods to deal with fault characteristics and human intelligent algorithms to build diagnosis models.

### 5.2. Discussion on Future Development

This paper summarizes the application of hydraulic pump fault research, but there are some inevitable omissions. To sum up, through the statistical analysis of the selected documents, it can be concluded that in the actual environment, it is difficult to obtain high-quality fault data from a single signal and extract the fault information contained. On the contrary, the multi-signal method is useful because it contains more information. The artificial intelligence method is useful because it has high feasibility in dealing with complex situations (such as compound faults). In order to better promote the development of hydraulic pump fault diagnosis, the following aspects can be carried out in the future:

(1) Because of the weak signal features in the early stage of fault, it is difficult to extract fault features, so fault feature extraction is still a direction that needs further exploration. Because of the powerful function of the deep learning method, fault feature extraction based on the deep learning method will be an important research direction.
(2) Although multi-data signals contain more information, the efficient information fusion methods for multi-data signals are still insufficient, so more efficient information fusion methods are also the direction to be further explored.
(3) From the statistical analysis of the review papers, it can be concluded that the diagnosis method of artificial intelligence will become mainstream. However, each intelligent method also has defects, and the combination of multiple intelligent methods can be used to fill the defects, such as reverse neural networks combined with multilayer perceptrons.

## 6. Conclusions

Fault diagnosis is the key to the health management of hydraulic pumps. It can improve the reliability of the hydraulic pump from the aspect of the data signal, and significantly reduce the risk of operation collapse and catastrophic failure. In recent years, the research on hydraulic pump fault diagnosis has been very active, but there is a lack of systematic analysis and summary of the developed methods. In order to make up for this gap, this paper systematically summarizes the relevant methods from the two aspects of fault diagnosis and health management. Finally, through the statistical analysis of the literature, some development prospects in this field are pointed out, which provides reference and guidance for researchers and practitioners to further carry out and apply relevant research. Nowadays, with the rapid development of machine learning algorithms and deep learning, data and signal-based methods are becoming the main direction in the future. The same trend applies to feature extraction methods. Therefore, the powerful ability of machine learning algorithms, especially deep learning algorithms, obviously has great potential in the future.

## Figures and Tables

**Table 1:** Wear classification.

Wear Type	Form Factor
Friction wear	The surface of the parts after manufacturing is always uneven when carefully observed with a magnifying glass. After the operation wear of the hydraulic pump, the metal particles fall off from the surface of the parts, and the uneven parts on the surface of the parts are relatively smoothed. If friction is continued later, deep marks or small-size wear will be produced. This kind of wear is normal natural friction wear.
Abrasive wear	According to the analysis of oil pollutants used in hydraulic pumps, more than 20% of the pollution particles are silica and metal oxides. These abrasive particles are the most serious components of pump parts wear. They are sandwiched between the surfaces of moving pair parts. When moving, they act as grinding sand, resulting in severe abrasive wear.
Pit wear	This is a kind of fatigue damage to hydraulic components. Under the action of alternating load, due to periodic compression and deformation, residual stress and metal fatigue will occur, resulting in tiny cracks on the parts, which will gradually cause small pieces of parts to peel off.
Corrosive wear	The surface of the hydraulic pump components is subjected to corrosive substances such as acids and moisture in the oil, and the metal surface is gradually damaged.

**Table 2:** Fault diagnosis method based on a single signal.

Year	Faults Studied				Signal Used	Method Used	Index Evaluation				Reference
	Fault I	Fault II	Fault III	Fault IV			Index I	Index II	Index III	Index IV	
2005			√		Vibration	WT+MRA (multi-resolution analysis)	√			√	[50]
2006	√		√		Vibration	fuzzy logic principle+Spectrum analysis			√		[84]
2008	√				Vibration	RNS+SVM		√			[75]
2008		√	√		Vibration	WA+PCA	√		√		[69]
2008	√			√	Vibration	PSD+DT+FIS	√	√			[86]
2009	√		√		Vibration	WPD			√		[41]
2011	√		√		Vibration	PCA				√	[52]
2011	√		√		Vibration	CPRBF		√		√	[67]
2011		√	√		Sound	KPCA			√		[87]
2012	√		√		Vibration	WP+MTS		√			[53]
2012	√		√		Vibration	SSSVN	√	√			[77]
2013		√		√	Vibration	EMD+NN	√		√		[56]
2013	√			√	Vibration	Spectrum analysis + rough set theory			√		[85]
2014	√	√			Vibration	PARD-BP		√			[70]
2014		√	√		Vibration	WPT+SOM		√		√	[59]
2015	√	√			Vibration	WPT+SVD+SVM	√		√		[72]
2015		√	√		Vibration	RELU-Dropout+SAE		√			[68]
2015	√		√		Vibration	LMD+Softmax	√			√	[58]
2015			√		Vibration	SIE+FCM		√			[83]
2015	√		√		Vibration	SOMP+compressive sensing theory		√			[54]
2015	√				Vibration	LMD+IAMMA	√	√			[48]
2015	√		√		Vibration	EMD+CEEMD+STFT+TFE+SVM	√		√		[76]
2015		√	√		Vibration	DCT+CNC+HHT	√		√		[38]
2016	√		√		Vibration	ITD+Softmax				√	[57]
2016	√	√		√	Vibration	7-layer CNN		√			[63]
2016		√	√		Vibration	HFHLCSD+BSS+DCTS+DCTHSE	√		√		[39]
2016		√	√		Vibration	WNC+CNN+HHT	√		√		[42]
2016	√				Vibration	ILCD+MF+BT-SVM	√		√		[78]
2017	√		√		Vibration	sensitivity analysis+PNN	√			√	[69]
2017			√		Vibration	EEMD+GA+SVR		√		√	[73]
2018		√			Vibration	LCD+DCS	√				[51]
2018			√		Vibration	SPIP+HMM		√			[43]
2018	√		√		Vibration	WPA+FE+LLTSA+SVM	√	√			[74]
2018	√	√			Vibration	WPT+LTSA+EMD+LMD+ELM				√	[82]
2019	√				Vibration	EWT+VCR	√		√		[35]
2019	√				Vibration	EMMD+Teager	√				[46]
2019			√		Vibration	FFT			√		[71]
2019	√		√		Sound	MFCC+ELM	√	√			[88]
2019	√		√		Vibration	IEWT		√			[47]
2019	√		√		Vibration	MCS+RE	√				[49]
2020	√		√		Vibration	EWT+PCA+ELM	√		√		[80]
2020	√	√			Vibration	CWT+CNN	√	√			[60]
2020	√				Vibration	EWT+VCR+HT	√				[45]
2020		√			Vibration	PSE	√				[37]
2020		√	√		Pressure	FEMD+RE	√		√		[91]
2020		√	√		Vibration	CWT+CNN+T-DSNE	√	√			[66]
2021	√				Vibration	MEEMD+AR+WKELM	√	√			[79]
2021	√		√		Vibration	CEEMDAN+CMBSE+t-SNE+WOA-KELM		√	√	√	[81]
2021	√		√		Vibration	WPA+AlexNet-CNN		√			[61]
2021	√		√		Vibration	PSO-Improve-CNN	√	√			[64]
2021	√				Angular velocity	IAS+NST			√		[92]
2021	√		√		Vibration	EEMD+Pearson	√		√		[36]
2021	√		√		Vibration	RMS	√				[55]
2022	√		√		Vibration	NCNN+Bayes+BP		√			[62]
2022	√		√		Vibration	WT+Bayes+CNN		√			[65]
2022		√	√		Pressure	CWT+Bayes+CNN		√			[90]
2022		√	√		Sound	CNN+PSO		√			[89]

**Table 3:** Fault diagnosis method based on multiple signals.

Year	Faults Studied				Signal Used	Index Evaluation				Reference
	Fault I	Fault II	Fault III	Fault IV		Index I	Index II	Index III	Index IV	
2002			√		Information fusion technology	√				[98]
2010		√	√		Hierarchical clustering analysis	√		√		[99]
2011	√		√		Improved DS evidence theory and spatiotemporal information fusion		√	√		[100]
2012	√		√		D-S+DMM	√		√		[111]
2013		√	√		Clustering diagnosis algorithm based on ARPD	√	√			[101]
2013	√	√			MFAM+Transfer learning		√			[113]
2014		√	√		PNN				√	[105]
2014	√				RBF-NN	√			√	[104]
2015	√		√		EEMD+Bayes+NN	√	√			[93]
2017	√		√		DS evidence theory	√				[94]
2017		√	√		EMD+PNN	√			√	[107]
2019	√		√		Inverse gaussian model + Bayes optimization		√		√	[97]
2020	√			√	PCA	√				[95]
2020		√		√	STFT+FFT	√				[96]
2020	√		√		SVM+Multilayer Perceptron(MLP)	√				[109]
2020	√				Stochastic forest neural network				√	[110]
2020	√		√		Singular value decomposition + transfer learning	√	√			[112]
2020	√				Reliability analysis + Bayesian network		√			[102]
2021	√				CNN based on improved adaptive learning rate		√		√	[103]
2021		√	√		KPCA+PNN		√			[108]
2021		√	√		CNN+EWT+WISE-PaaS	√	√			[114]
2022	√		√		Wavelet packet analysis+PNN	√			√	[106]

## Data Availability

There is no any data involved.

## Nomenclature

AOPF	Adaptive-Order Particle Filter
AR	Autoregressive
BE	Bispectrum Entropy
BI-LSTM	Bi-Directional Long-Short Term Memory
BT-SVM	Binary Tree Support Vector Machine
CEEMD	Complementary Ensemble Empirical Mode Decomposition
CEEMDAN	Complete Ensemble Empirical Mode Decomposition with Adaptive Noise
CMBSE	Composite Multi-Scale Basic Scale Entropy
CNC	Cosine Neighboring Coefficients
CNN	Convolutional Neural Network
CPRBF-NN	Radial Basis Function Network In Conjunction With Chaos Theory
CWT	Continuous Wavelet Transform
DBN	Deep Belief Network
DCAE	Deep Convolutional Autoencoder
DCS	Discrete Cosine Transform–Composite Spectrum
DCT	Discrete Cosine Transform
DLDR	Double Linear Damage Rule
DT	Decision Trees
EEMD	Ensemble Empirical Mode Decomposition
ELM	Extreme Learning Machine
EM	Expectation Maximization
EMD	Empirical Mode Decomposition
EMMD	Extremum Field Mean Mode Decomposition
ESN	Modified Echo State Networks
EWT	Empirical Wavelet Transform
FA	Factor Analysis
FCM	Fuzzy C-Means
FE	Fuzzy Entropy
FEMD	Fast Empirical Mode Decomposition
FFT	Fast Fourier Transform
FIS	Fuzzy Inference System
FMEA	Failure Mode And Effects Analysis
FMECA	Modes, Effects, And Criticality Analysis
GA	Genetic Algorithm
HHT	Hilbert–Huang Transform
HMM	Hidden Markov Model
HT	Hilbert Transform
IAMMA	Improved Adaptive Multiscale Morphology Analysis
Ir-PCA	Informative ratio-Principal component analysis
IEWT	Improved Empirical Wavelet Transform
IG	Inverse Gaussian
ILCD	Improved Local Characteristic-Scale Decomposition
ITD	Intrinsic Time-Scale Decomposition
JITL	Just In Time Learning
KPCA	Kernel Principal Component Analysis
KS	Kolmogorov-Smirnov
K-VNN	K-Vector Nearest Neighbor
LCD	Local Characteristic-Scale Decomposition
LLTSA	Liner Local Tangent Space Alignment
LMD	Local Mean Decomposition
LR	Logistic Regression
LTSA	Local Tangent Space Alignment
MAAKR	Modified Auto-Associative Kernel Regression
MCPF	Monotonicity-Constrained Particle Filtering
MCS	Monte Carlo Simulation
MD	Mahalanobis Distance
MEEMD	Modified Ensemble Empirical Mode Decomposition
MF	Multi-Fractal Spectrum
MFAM	Multi-Signal Fusion Adversarial Model
MFCC	Mel-Frequency Cepstral Coefficient
MHAPE	Modified Hierarchical Amplitude-Aware Permutation Entropy
MLE	Maximum Likelihood Estimation
MLP	Multilayer Perceptron
MMPE	Mean Of Multi-Scale Permutation Entropy
MPE	Multi-Scale Permutation Entropy
MTS	Mahalanobis–Taguchi System
NCNN	Normalized Convolutional Neural Network
NE	Norm Entropy
NN	Neural Network
PARD	Pruning Algorithm Based Random Degree
PCA	Principal Component Analysis
PCC	Pearson Correlation Coefficient
PHM	Prognostics And Health Management
PNN	Probabilistic Neural Network
PSD	Power Spectral Density
PSE	Power Spectral Entropy
PSO	Particle Swarm Optimization
QPSO	Quantum Particle Swarm Optimization
RBF	Radial Basis Function
RBM	Boltzmann Machine
RE	Relative Entropy
RFC	The Random Forest Classifier
RMS	Root Mean Square
RNS	Real-Valued Negative Selection
RSDD	Resonance-Based Sparse Signal Decomposition
RUL	Remaining Useful Life
SAE	Stacked Autoencoders
SEOS	Smoothed Energy Operation Separation
SIE	Spatial Information Entropy
SMOTE	Synthetic Minority Over-Sampling Technique
SOM-NN	Self-Organizing Mapping Neural Network
SOMP	Stagewise Orthogonal Matching Pursuit
SPIP	Symbolic Perceptually Important Point
SS-SVM	Sphere-Structured Support Vector Machines
STFT	Short Time Fourier Transform
SVD	Singular Value Decomposition
SVM	Support Vector Machine
SVR	Support Vector Regression
T-DSNE	T-Distributed Stochastic Neighbor Embedding
TFE	Time-Frequency Entropy
T-SNE	T-Distributed Stochastic Neighbor Embedding
VCR	Variance Contribution Rate
VMD	Variation Mode Decomposition
WA	Wavelet Analysis
WCRA	Wavelet Coefficient Residual Analysis
WKELM	Wavelet Kernel Extreme Learning Machine
WNC	Wavelet Neighboring Coefficients
WOA	Whale Optimization Algorithm
WOA-KELM	Whale Optimization Algorithm Kernel Extreme Learning Machine
WP	Wavelet Packet
WPA	Wavelet Packet Analysis
WPD	Wavelet Packet Decomposition
WPNE	Wavelet Packet Norm Entropy
WPT	Wavelet Packet Transform
WT	Wavelet Transform
